# Phytol in Skin Care: From Multidimensional Pharmacological Mechanisms to Nanocarrier-Based Cosmetic Applications

**DOI:** 10.3390/ijms27177660

**Published:** 2026-08-26

**Authors:** Xiaohan Wu, Wenxiang Zhang, Bohao Jin, Siyu Chen, Hong Shen

**Affiliations:** 1 State Key Laboratory of Natural Medicines, School of Life Science and Technology, China Pharmaceutical University, Nanjing 211198, Chinawenxiangzhang@cpu.edu.cn (W.Z.);; 2 Yunnan Characteristic Plant Extraction Laboratory, Kunming 650106, China

**Keywords:** phytol, skincare, skin pharmacological mechanism, nanodelivery systems, cosmetic application

## Abstract

Phytol, an acyclic diterpene alcohol and a key lipophilic side-chain moiety of chlorophyll, is widely distributed in nature. It exhibits potent antioxidant, anti-inflammatory, analgesic and broad-spectrum antibacterial activities. Notably, phytol can also effectively inhibit melanin production, repair the skin barrier, and exert profound anti-aging effects, making it a highly promising ingredient for daily skincare with substantial industrial application value. The skincare benefits of phytol are primarily achieved by constructing a multi-dimensional regulatory network involving defense, modulation and repair. Compared to conventional retinol-based skincare ingredients, phytol exhibits superior biocompatibility, mild irritation, and remarkable safety advantages. Nevertheless, its application is hindered by inherent limitations, including strong hydrophobicity, spontaneous aggregation tendency, and poor photothermal stability. These drawbacks severely restrict its dispersibility, storage stability and percutaneous bioavailability in aqueous cosmetic formulations. To address these deficiencies, nanodrug delivery systems (NDDS), such as liposomes, nanoemulsions, solid lipid nanoparticles and PLGA nanoparticles, have been widely employed. These nanocarriers can penetrate the skin barrier via the size effect, enabling targeted skin delivery and long-term controlled release of phytol. This review systematically summarizes the biological sources and metabolic fate of phytol, as well as its multi-mechanistic pharmacological effects on the skin. Furthermore, we outline the current application status and industrial development trends of phytol in mainstream cosmetics worldwide. This work aims to provide theoretical basis and forward-looking references for the development of high-efficiency, safe and stable phytol-derived skincare raw materials and topical formulations. Highlights: (1) Phytol, a natural acyclic diterpene alcohol, exerts multi-dimensional skincare effects including antioxidant, anti-inflammatory, whitening, anti-aging, and skin barrier repair activities via a defense–modulation–repair regulatory network. (2) Phytol may act as a mild, non-irritating functional alternative to retinoids, targeting PPAR/RXR pathways and avoiding TRPV1-mediated irritation, making it suitable for sensitive skin. (3) Poor water solubility and instability hinder phytol’s translation; nanodelivery systems effectively improve solubility, permeability, and sustained release.

## 1. Introduction

**Figure 1:**
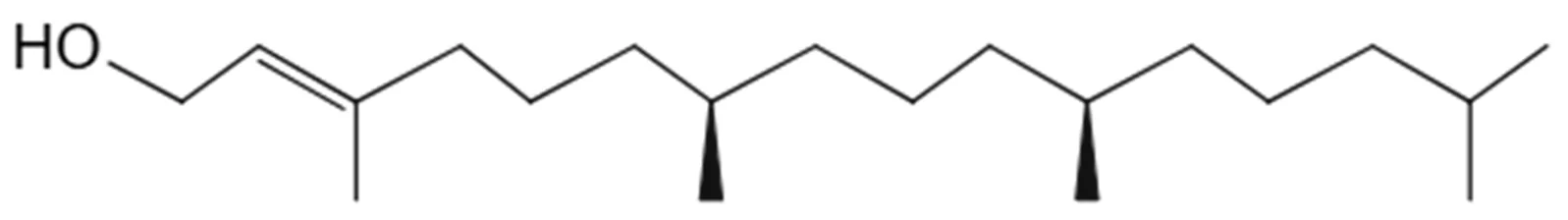
Chemical structure of phytol.

As consumers increasingly pursue the “Clean Beauty” and “Efficiency Skincare”, finding highly active, low-irritation alternative ingredients from natural biological resources has become a main trend in cosmetic research and development [1]. Phytol is a naturally occurring acyclic diterpene alcohol and an indispensable lipophilic side-chain structure of chlorophyll (Figure 1) [2,3]. Since its first isolation in 1909 and subsequent confirmation as a structural component of chlorophyll [4,5], phytol has drawn continuous attention from academia and industry, showing promising application prospects in pharmaceuticals, food and daily skincare fields.

At the biological level, phytol not only serves as a key precursor for the synthesis of vitamins E and K1 but also plays an irreplaceable role in plant stress resistance and animal metabolism [6]. Recent pharmacological studies have further revealed its multifaceted potential in skincare. As a potent antioxidant, phytol counteracts oxidative stress through both chemical scavenging and biological modulation. In addition, it exhibits significant anti-inflammatory, analgesic, and broad-spectrum antimicrobial properties, making it highly effective for soothing sensitive skin and improving problematic skin conditions [7]. Notably, phytol serves as a mild putative retinoid-like precursor that may promote skin-barrier repair and confer anti-aging effects with reduced risk of the irritation seen with conventional retinoids. Nevertheless, direct evidence supporting the metabolic conversion of phytol to phytanic acid within human skin remains limited, and its effects are putatively mediated via activation of nuclear receptor signaling pathways such as PPARs/RXRs [8,9]. Furthermore, its ability to inhibit melanogenesis and melanoma cell proliferation provides a solid basis for applications in skin brightening and spot reduction [10].

Nevertheless, the effective incorporation of phytol into cosmetic products faces serious physicochemical challenges. Owing its high hydrophobicity, phytol exhibits poor water dispersion and tends to aggregate in aqueous solutions. Moreover, it is prone to oxidation and degradation when exposed to light or heat. These issues severely restrict its bioavailability and formulation stability [11,12,13]. To overcome these limitations, nanoscale delivery systems, such as liposomes, nanoemulsions, and solid lipid nanoparticles, have been introduced for phytol encapsulation. These nanocarriers not only enhance the solubility and chemical stability of phytol but also, due to their nanoscale characteristics, improve skin permeability and enable targeted and controlled release within the skin [14].

This review systematically summarizes the biological sources, metabolic profiles, pharmacological activities, and nanocarrier delivery strategies of phytol. Additionally, we analyze the current application status and market development trends of phytol in mainstream cosmetics. The aim is to provide a reference for the development of efficient, safe and stable delivery systems, as well as high-performance skincare formulations.

## 2. Methods

Electronic literature searches were performed in PubMed and Web of Science Core Collection, among other bibliographic databases. Search keywords comprised phytol combined with antioxidant, anti-inflammatory, skin-related activity, pharmacological effect, mechanism, nanocarrier, delivery system, cosmetic application, and other related terms. Publicly accessible resources from the COSING cosmetic-ingredient database were also consulted. Conference abstracts, editorials, and patent documents were excluded. Duplicate entries were first removed, followed by title-and-abstract screening and subsequent full-text assessment to filter eligible references for this review. Reference lists of eligible articles were further manually screened to identify additional relevant records.

## 3. Source, Biosynthesis and Metabolic Degradation Pathway of Phytol

Phytol (chlorophyll alcohol) is an acyclic isoprenoid compound with the molecular formula C_20_H_40_O and represents one of the most abundant naturally occurring diterpenoids [15,16]. As an essential structural component of chlorophyll, phytol is widely present in almost all photosynthetic organisms, including higher plants, algae, and cyanobacteria [7].

### 3.1. Biosynthetic Pathway of the Phytol Skeleton

**Figure 2:**
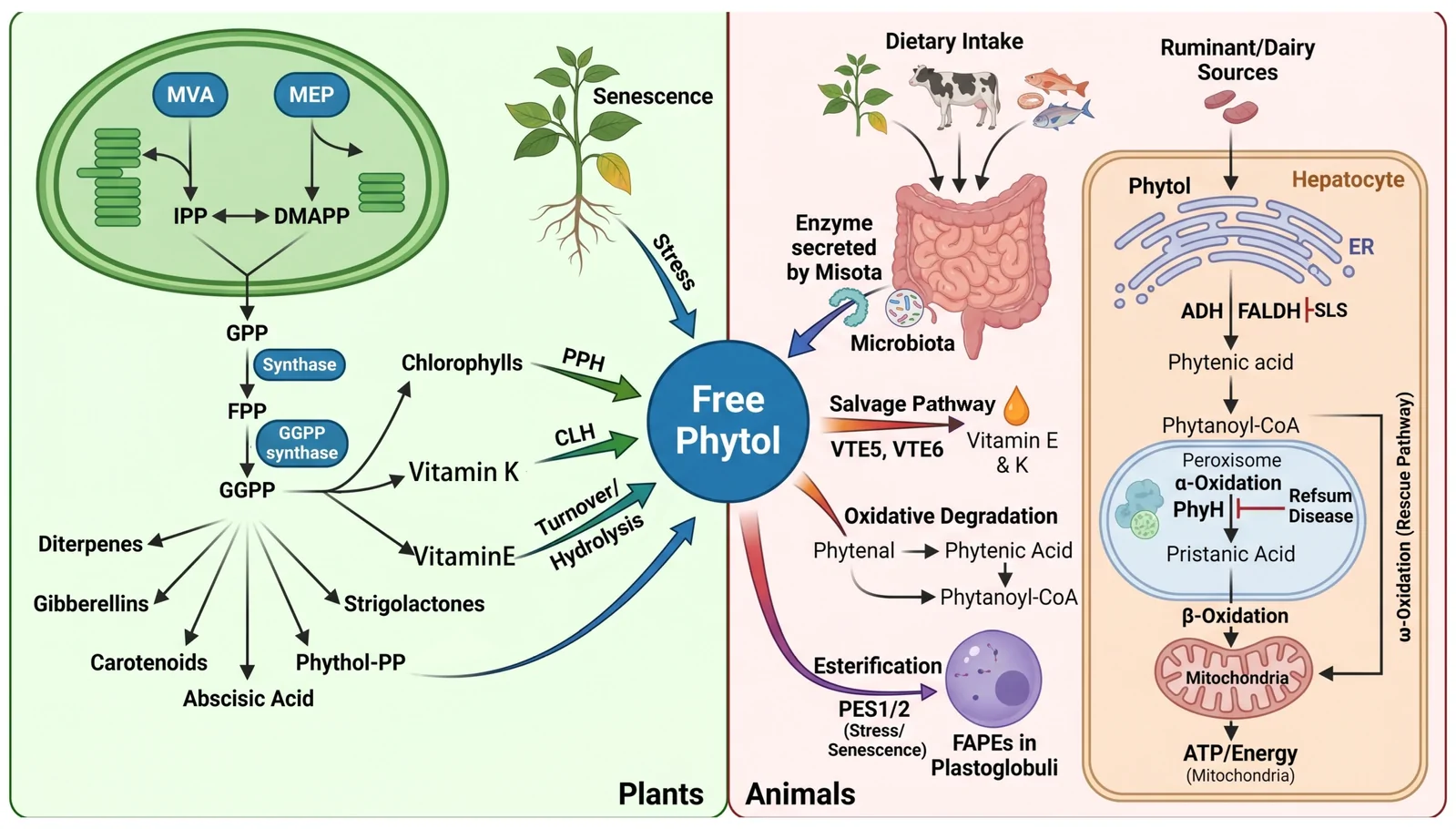
Biosynthetic pathway and metabolic fate of phytol in plants and mammals. Plant-derived free phytol is generated from chlorophyll degradation and turnover under stress and senescence [2,3,24,25,26,27,28,29,30,31,32,33,34,35]. In animals, dietary phytol is processed by gut microbiota, and undergoes multiple metabolic routes including vitamin E/K salvage [17,18,36], oxidative degradation [13,37,38], esterification [39,40], and hepatic peroxisomal-mitochondrial catabolism for energy production [38,41,42,43,44,45,46,47]. The ω-oxidation rescue pathway is involved in Refsum disease [41,46]. The left-hand green panel shows plant biosynthetic compartments (plastids for MEP and MVA pathways); the right-hand beige panel depicts hepatic and cellular metabolic compartments in animals.

Phytol is biosynthesized in plants through the methylerythritol phosphate (MEP) pathway in plastids (Figure 2), which provides the isoprenoid precursor for chlorophyll side chain formation [17,18]. It is widely distributed in various photosynthetic plants, with relatively high content in green tea, mulberry leaves, perilla and other medicinal and edible plants [19,20,21]. Industrially, phytol is mainly obtained through alkaline hydrolysis and solvent extraction from chlorophyll-rich raw materials such as silkworm excrement and green tea residues [22,23,24], and can also be synthesized via chemical reduction of isophytol or citral [25,26]. However, from the perspective of cosmetic raw material supply, the detailed plant biosynthetic pathway is less relevant to skincare applications, and natural extraction remains the dominant route for cosmetic-grade phytol due to consumer preference for natural-sourced ingredients and the relatively high cost of chemical synthesis [27,28].

### 3.2. Natural Sources and Industrial Preparation

In nature, phytol exists mainly as the esterified side chain of chlorophyll, and is released during chlorophyll degradation in plant tissues [29,30]. Commercially, phytol is primarily obtained through alkaline hydrolysis of chlorophyll-rich plant materials, including mulberry leaves, silkworm excrement, green tea residues and other agricultural by-products [31,32,33]. Alternatively, phytol can be chemically synthesized via reduction of isophytol or citral, with different synthetic routes exhibiting distinct advantages in yield and production cost [34,35]. However, natural extraction remains the dominant route for cosmetic-grade phytol due to growing consumer preference for natural-sourced ingredients and the quality requirements of high-end skincare formulations [48,49,50].

### 3.3. Metabolic Fate of Free Phytol in Plants

In plant cells, free phytol functions as a key metabolic node whose fate is regulated by developmental and environmental signals [51]. It is primarily metabolized through three pathways: phosphorylation for reutilization, esterification for storage, and oxidative degradation for energy recovery. Via the salvage pathway, free phytol is sequentially phosphorylated to phytyl diphosphate, which serves as the side-chain precursor for tocopherol and phylloquinone biosynthesis [36]. Under stress conditions, excess free phytol is esterified with fatty acids to form fatty acid phytyl esters (FAPEs), which are stored in plastoglobuli as a detoxification and reservoir mechanism (Figure 2) [39,40]. Alternatively, phytol can be oxidized to phytanoyl-CoA and further degraded via peroxisomal α-oxidation followed by β-oxidation to recover carbon and energy (Figure 2) [37,38].

### 3.4. Metabolic Fate of Phytol in Animals and Humans

Unlike plants, mammals including humans cannot synthesize phytol endogenously, and all body phytol originates from exogenous dietary intake, mainly from meat and dairy products derived from ruminants [41,52]. Following intestinal absorption, phytol is primarily transported to the liver for stepwise metabolic degradation [52,53]. Due to the methyl branch at the β-carbon of its side chain, phytol and its metabolites cannot directly enter mitochondrial β-oxidation, and must undergo a specialized multi-step pathway including oxidation, activation, reduction by trans-2-enoyl-CoA reductase (TER) and peroxisomal α-oxidation before entering conventional β-oxidation for complete breakdown (Figure 2) [42,43,44].

Genetic defects in key enzymes of this metabolic pathway lead to severe clinical disorders. Loss-of-function mutations in *ALDH3A2* encoding fatty aldehyde dehydrogenase (FALDH) cause Sjögren-Larsson syndrome, characterized by neurocutaneous symptoms due to impaired long-chain fatty aldehyde metabolism including phytenal [45]. Deficiency of phytanoyl-CoA hydroxylase (PhyH), the rate-limiting enzyme of α-oxidation, results in Refsum disease, with pathological accumulation of phytanic acid and severe manifestations such as retinitis pigmentosa and polyneuropathy [46]. Notably, the understanding of this pathway has evolved: early studies proposed direct reduction of phytenic acid [47], while subsequent metabolomic research established the “activation-then-reduction” model via phytenoyl-CoA intermediate [54].

## 4. Research Progress on the Potential Skincare Mechanisms of Phytol

### 4.1. Fundamental Defense Mechanisms: Antioxidant Defense, Analgesia, and Antipruritic Effects

**Figure 3:**
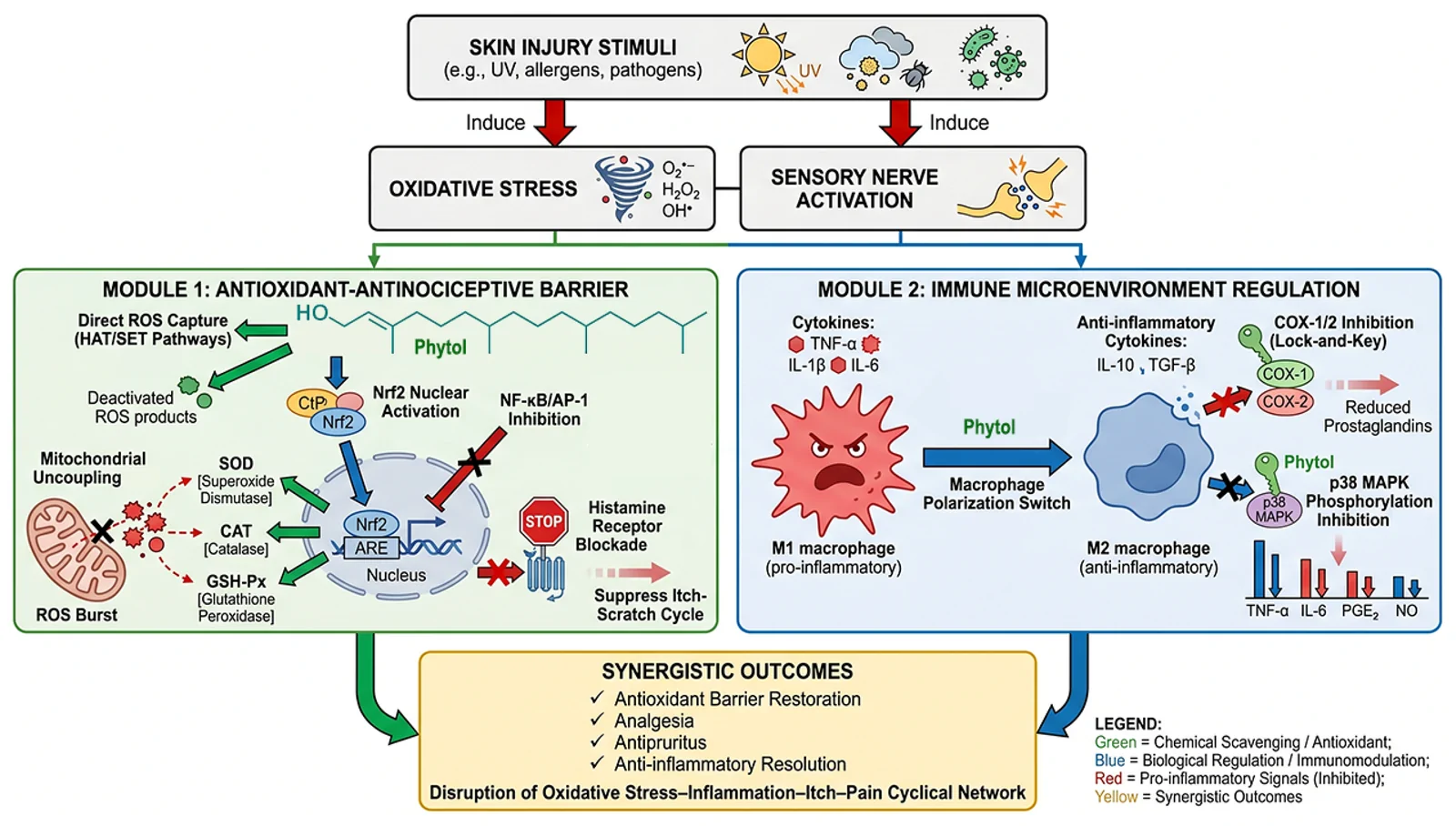
Schematic illustration of phytol’s dual-modulation mechanisms against skin injury stimuli. Protective effects of phytol against skin-injury-triggered oxidative stress and sensory-nerve activation. Module 1 depicts the antioxidant-antinociceptive barrier: phytol directly scavenges ROS [5,55,56,57], activates the Nrf2-ARE pathway to boost antioxidant-enzyme expression [56,58,59,60], inhibits NF-κB/AP-1, and blocks itch-scratch cycles [56,58,59]. Module 2 shows immune-microenvironment modulation: phytol promotes M1-M2 macrophage polarization, suppresses pro-inflammatory mediators, and inhibits COX-1/2 and p38 MAPK signaling [7,60,61,62,63,64]. Symbol definitions: solid arrows denote activation/induction; crossed-block blunt arrows represent molecular inhibition. Color coding follows the embedded in-figure legend: green = chemical-scavenging/antioxidant events; blue = biological-regulation/immunomodulation; red = pro-inflammatory signals that are suppressed; yellow = integrated synergistic biological outcomes.

Oxidative stress and pain sensitization serve as early biomarkers of skin injury. Phytol establishes a dual “antioxidant-analgesic” defensive barrier, thereby providing primary cellular-level protection to the skin (Figure 3).

#### 4.1.1. The Antioxidant Activity of Phytol

The antioxidant efficacy of phytol has been well documented across various in vitro and in vivo models (Figure 3). At the biochemical level, phytol demonstrates dose-dependent DPPH radical-scavenging activity and effectively restores intracellular glutathione (GSH) homeostasis [58]. In cellular models of oxidative stress, phytol suppresses reactive oxygen species (ROS) generation by activating the Nrf_2_/HO-1 signaling pathway, thereby inhibiting RANKL-induced osteoclast differentiation [59]. Furthermore, phytol exhibits dual protective functions in neuronal cell models, i.e., antioxidant and cholinesterase-inhibitory activities [65]. In biochemical assays, phytol exhibited 59.89 ± 0.73% DPPH radical-scavenging activity at 7.2 µg/mL, with an EC_50_ of 4.87 µg/mL [55]. These functions are reinforced by in vivo studies, demonstrating that phytol can effectively scavenge free radicals and enhance the endogenous antioxidant defense system [55,56]. Moreover, phytol often acts synergistically with other bioactive constituents in plant extracts, contributing to a coordinated antioxidant network [66]. Current mechanistic studies indicate that the antioxidant properties of phytol are primarily mediated by two complementary pathways: direct chemical radical scavenging and modulation of cellular antioxidant signaling (Figure 3).

One is chemical scavenging mechanisms (HAT and SET Pathways). The HAT pathway serves as the predominant chemical mechanism responsible for the direct radical-scavenging capacity of phytol. For DPPH• radical elimination, phytol primarily operates via the HAT mechanism. As a branched-chain unsaturated alcohol, phytol possesses an allylic hydroxyl group that acts as a critical hydrogen-donating structural moiety [56]. In DPPH assays, the allylic hydroxyl moiety of phytol readily donates a hydrogen atom (H•) to DPPH radicals. Hydrogen abstraction generates an intermediate carbon-centered radical stabilized by resonance conjugation with the adjacent C=C double bond, which lowers the activation energy of the reaction [55]. When exposed to reactive oxygen species and free radicals, including DPPH• and hydroxyl radicals (•OH), phytol actively donates hydrogen atoms to neutralize and convert radical species into stable products. Meanwhile, phytol itself is transformed into a relatively stable radical intermediate, thereby effectively terminating the oxidative chain reaction and alleviating oxidative damage [67].

**Figure 4:**
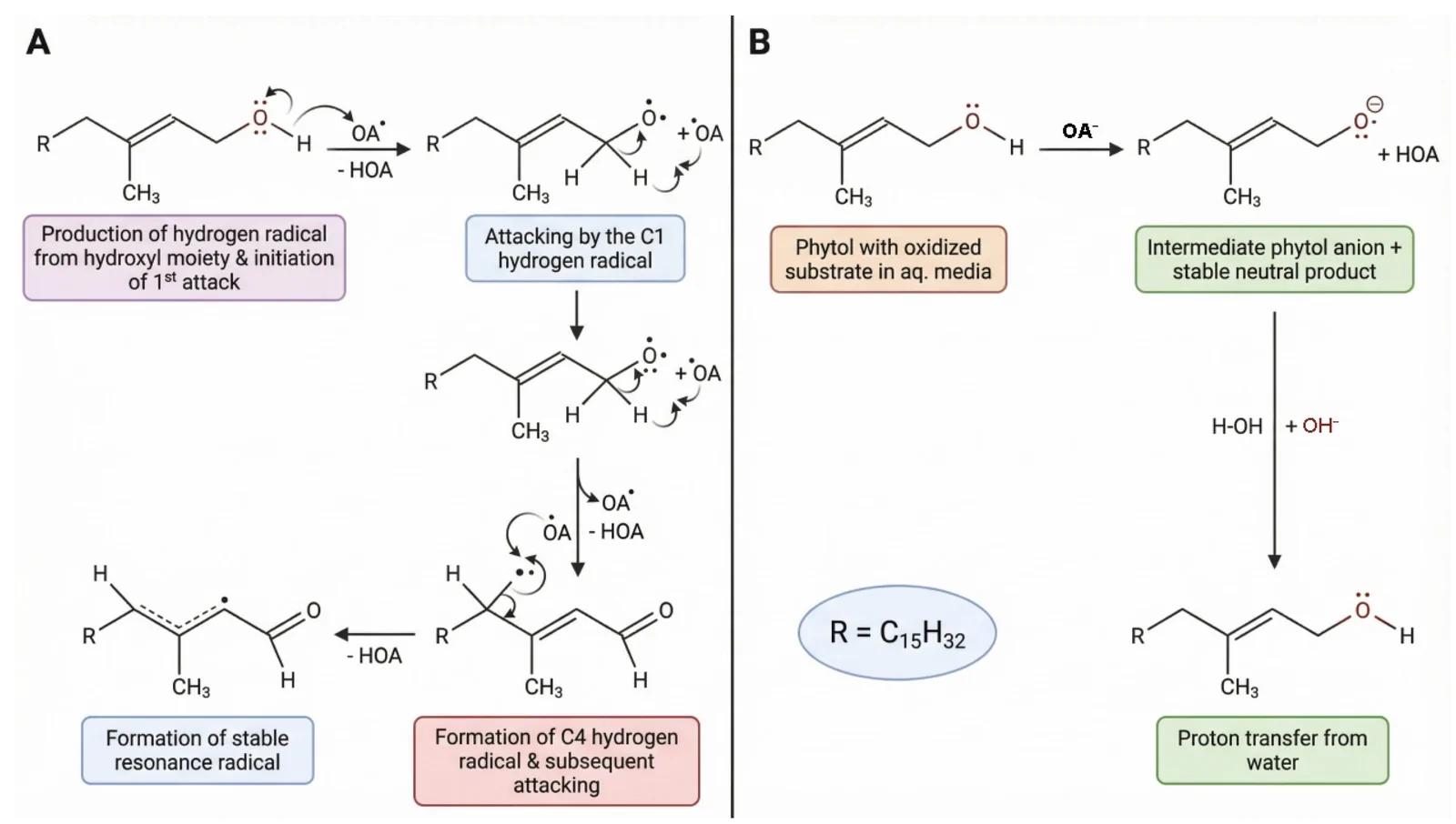
Hydrogen-atom transfer (HAT) pathway and single-electron transfer (SET) pathway of phytol-mediated antioxidation. (**A**) HAT: Hydrogen-atom transfer pathway. (**B**) SET: Single-electron transfer pathway. Solid arrows trace hydrogen-and electron-transfer trajectories; colored boxes denote reactive intermediate radical species. R = C_15_H_32_ corresponds to the alkyl hydrocarbon tail moiety of phytol; OA• represents oxidative radical acceptors.

The SET pathway is another essential chemical antioxidant mechanism. Owing to its low ionization energy, phytol can function as an electron donor. In polar solvent environments and physiological conditions, phytol transfers a single electron to electron-deficient oxidants and radical cations, leading to rapid radical neutralization. Subsequently, phytol is converted into a cation radical and further stabilized through deprotonation and secondary structural rearrangement, thereby achieving continuous antioxidant effects (Figure 4) [57].

Another is biological regulatory mechanisms. Phytol exerts its antioxidant effects through dual biological pathways. First, it induces a mild “uncoupling effect” on mitochondrial membrane potential, reducing endogenous ROS production at the source (Figure 3). Second, it activates the Nrf2/ARE signaling pathway, upregulating the expression of key antioxidant enzymes such as SOD and CAT (Figure 3) [55,59,60]. Given that oxidative stress is a significant trigger of inflammatory responses, phytol effectively disrupts the vicious cycle of “oxidative stress–inflammation” by enhancing the activity of endogenous antioxidant enzymes (e.g., SOD, GSH-Px) and lowering ROS levels.

Nevertheless, it should be noted that direct FRAP assay data for purified phytol monomer are still scarce in available literature. Although plant extracts containing phytol exhibit obvious ferric-reducing antioxidant capacity in FRAP tests, such activity arises from the synergistic effect of multiple co-existing antioxidant ingredients rather than phytol alone [68]. Further FRAP evaluation using purified phytol is required to achieve a more comprehensive understanding of its antioxidant potential.

#### 4.1.2. Antinociceptive and Antipruritic Effects of Phytol

Phytol exhibits notable activity in alleviating skin sensory nerve discomfort, e.g., pain and pruritus. Its action mechanism is closely linked to its potent antioxidant and anti-inflammatory properties (Figure 3). In vivo studies have confirmed that phytol possesses significant antinociceptive activity [56], showing favorable application potential for soothing sensitive skin and managing inflammatory skin disorders based on animal experimental evidence and skin safety evaluation data.

At the molecular level, phytol exerts its antinociceptive and antipruritic effects primarily through multi-targeted regulation of inflammatory mediators. Specifically, it effectively inhibits the NF-κB signaling pathway, leading to the downregulation of key pro-inflammatory cytokines such as TNF-α and IL-6 [60], and suppresses prostaglandin E2 (PGE2) release (Figure 3). This attenuation of the inflammatory cascade not only produces direct analgesic effects but also establishes a favorable low-inflammatory microenvironment conducive to tissue repair [69]. Further studies have demonstrated that phytol exhibits significant anti-scratching activity in the context of allergic skin disorders, such as atopic dermatitis (AD). In mouse models induced by histamine and compound 48/80, phytol effectively suppressed scratching behavior, with its efficacy positively correlated with its ability to inhibit vascular permeability [61]. Mechanistic analysis revealed that, in addition to the NF-κB pathway, phytol also inhibits the activation of the transcription factor AP-1, thereby downregulating the expression of allergy-associated cytokines (e.g., IL-4) and TNF-α [61]. Thus, by synergistically suppressing the transcriptional activity of NF-κB/AP-1 and the release of downstream inflammatory/allergic mediators, phytol disrupts the “inflammation–itch–pain” cycle at the molecular level, offering a promising therapeutic strategy for soothing and repairing sensitive and allergic skin.

### 4.2. Regulating the Immune Microenvironment: Multi-Targeted Anti-Inflammatory Mechanisms

In the context of anti-inflammatory pharmacology, phytol demonstrates significant therapeutic potential through its multi-dimensional modulation of inflammatory mediators. The mechanisms encompass both cellular immunomodulation and targeted interference with key signaling pathways.

At the cellular and immunoregulatory level, phytol exhibits distinct modes of action depending on the inflammatory phase (Figure 3). In acute inflammation models, phytol primarily attenuates the inflammatory cascade by suppressing neutrophil infiltration, which is associated with decreased levels of pro-inflammatory cytokines such as IL-1β and TNF-α, as well as reduced oxidative stress markers [70]. During chronic inflammation, phytol modulates immune homeostasis by reshaping the functional plasticity of immune cells. Experimental evidence indicates that phytol can influence the polarization of monocyte-derived macrophages, promoting a shift toward an anti-inflammatory phenotype, thereby contributing to the resolution of sustained inflammatory responses [60,71].

At the molecular signaling pathway level, phytol exerts its anti-inflammatory effects primarily through the dual inhibition of the NF-κB and MAPK signaling cascades, suppressing the release of pro-inflammatory mediators (Figure 3). Specifically, phytol impedes the phosphorylation and subsequent degradation of IκBα, which prevents the nuclear translocation of the NF-κB p65 subunit and attenuates the transcriptional activation of key pro-inflammatory cytokines, including IL-6, TNF-α, and IL-1β [60,62]. In parallel, phytol downregulates the phosphorylation of p38 MAPK, further inhibiting inflammatory signal transduction [63]. Notably, given that oxidative stress serves as a critical driver of inflammatory responses, phytol also disrupts the self-perpetuating “oxidative stress–inflammation” cycle by enhancing the activity of endogenous antioxidant enzymes such as superoxide dismutase (SOD) and glutathione peroxidase (GSH-Px), while concurrently reducing intracellular reactive oxygen species (ROS) levels.

Molecular docking and computational simulation studies have identified the direct molecular targets of phytol (Figure 3). Modeling results demonstrate that phytol forms stable binding interactions with cyclooxygenase-1 (COX-1), cyclooxygenase-2 (COX-2), NF-κB, and IL-1β. This indicates that phytol exerts a dual inhibitory effect on inflammatory mediator synthesis: it not only indirectly downregulates COX enzyme expression by suppressing upstream IL-1β and NF-κB signaling, but also directly inhibits the enzymatic activity of COX-1/2, thereby blocking the production of prostaglandins and other inflammatory mediators [7,64].

Phytol also exerts synergistic anti-inflammatory effects when combined with other natural ingredients. For example, in a mouse skin model of *Candida albicans* infection, phytol synergized with other components in *Spirulina maxima* extract to significantly attenuate oxidative stress-induced inflammatory responses, effectively alleviating skin erythema, swelling, and hyperalgesia [72]. In conclusion, phytol exerts anti-inflammatory effects through a multi-target, multi-pathway mechanistic network.

### 4.3. Antimicrobial Activity of Phytol

**Figure 5:**
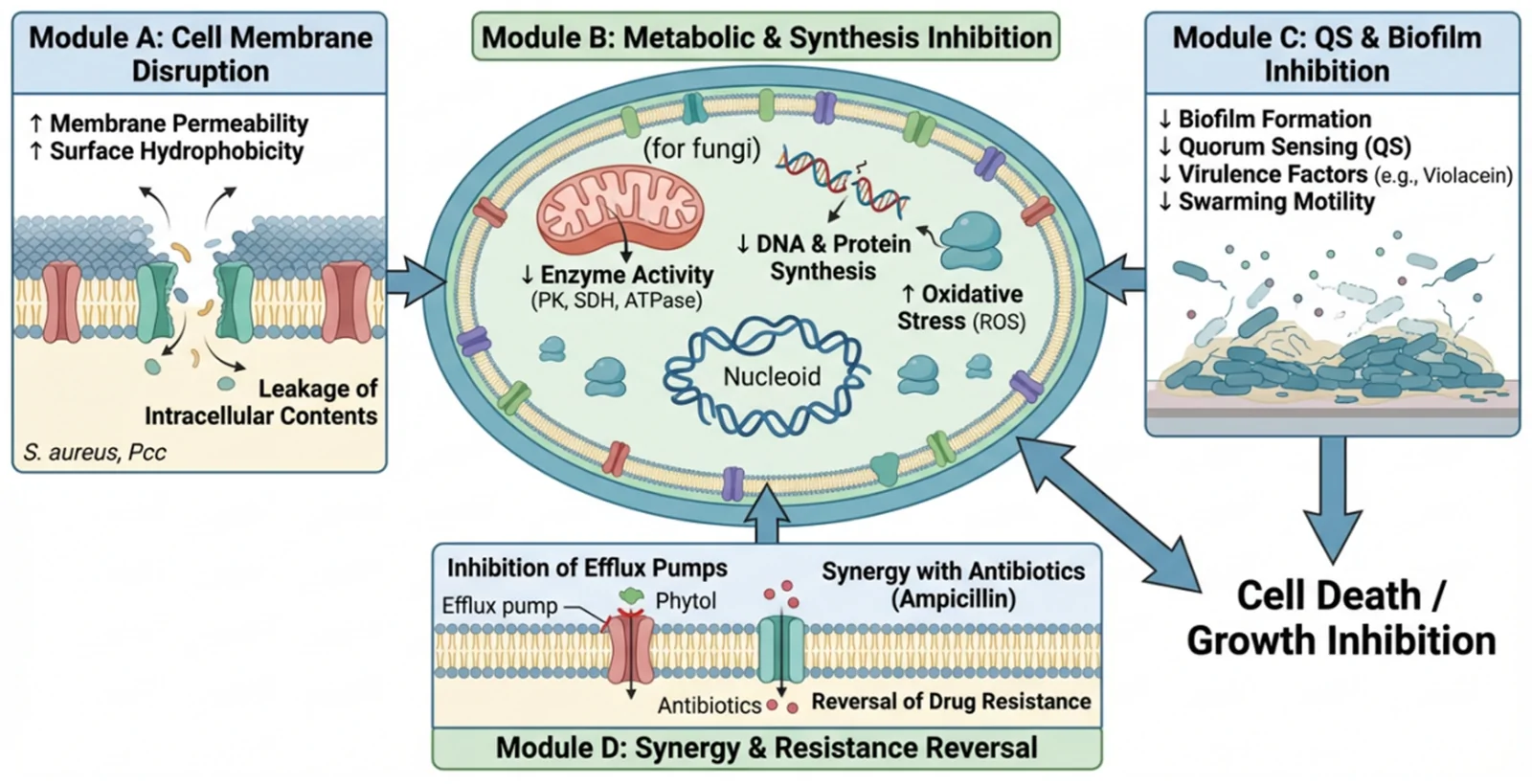
Multi-modal antibacterial mechanisms of phytol. (**A**) phytol disrupts bacterial membranes to induce intracellular-content leakage in *S. aureus* and *Pcc* [75,76,77]. (**B**) phytol inhibits metabolic enzymes, DNA/protein synthesis and elevates oxidative stress [76,78]. (**C**) phytol suppresses biofilm formation, quorum sensing, virulence factors and swarming motility [78]. (**D**) efflux-pump inhibition enables synergistic antibacterial activity with ampicillin and reverses drug resistance [78,79,80]. Symbol definitions: outward-pointing arrows indicate increased membrane permeability or substance leakage; inward-directed block arrows represent inhibitory actions on molecular targets. Each colored module box corresponds to one functional antibacterial mode of phytol.

Phytol exhibits potent broad-spectrum antimicrobial activity, inhibiting various bacteria and fungi, particularly pathogens associated with skin infections. Studies have demonstrated that its antimicrobial effects against *Escherichia coli*, *Candida albicans*, and *Aspergillus niger* are dose- and time-dependent, with a MIC_50_ value of 62.5 µg/mL for these three strains. By contrast, *Staphylococcus aureus* showed high tolerance to phytol under the tested conditions, with a MIC_50_ greater than 1000 µg/mL [73]. Regarding antifungal activity, phytol effectively suppresses common pathogenic fungi responsible for cutaneous infections, including *Candida albicans*, *Trichophyton rubrum*, and *Malassezia furfur*. In vivo experiments further confirm that topical application of phytol-loaded cream efficiently eradicates *Candida* spores within the skin layers and significantly ameliorates fungal-induced skin lesions [72]. Additionally, stevia extracts enriched in phytol have shown marked inhibitory activity against animal skin pathogens [74]. The antimicrobial activity of phytol is mediated by a multi-target synergistic mechanism, encompassing three core pathways: disruption of cell membrane structural integrity, interference with quorum sensing and biofilm formation, and blockade of key metabolic pathways (Figure 5).

First, disruption of cell membrane structure and function is the primary mechanism underlying phytol’s antimicrobial activity (Figure 5B). Phytol directly targets pathogen cell membranes by either altering lipid fluidity or binding to membrane proteins, thereby impairing membrane stability [75,76]. In studies on *Pectobacterium carotovorum* subsp. *carotovorum* (*Pcc*), phytol not only induces morphological changes in bacterial cells but also increases cell surface potential and hydrophobicity, leading to cell wall damage and loss of cell membrane integrity [75,76]. Similarly, studies on *Staphylococcus aureus* have confirmed that phytol significantly enhances cell membrane permeability, resulting in the leakage of intracellular contents [77].

Second, phytol effectively modulates the quorum sensing (QS) system and biofilm formation of pathogens, reducing their drug resistance and pathogenicity (Figure 5C). Studies have demonstrated that phytol significantly inhibits biofilm formation and reduces surface adhesion capacity in *Pseudomonas aeruginosa* [78]. Regarding QS regulation, phytol blocks QS signal transduction in *Chromobacterium violaceum*, diminishes the production of the virulence factor violacein, and suppresses bacterial swarming motility—collectively reducing the competitive survival advantage of pathogenic bacteria [78].

Third, phytol blocks pathogen proliferation by suppressing key metabolic enzyme activities and inducing oxidative stress (Figure 5B). In terms of energy metabolism, phytol significantly inhibits the activities of pyruvate kinase (PK), succinate dehydrogenase (SDH), and ATPase in *Pectobacterium carotovorum* subsp. *carotovorum* (*Pcc*), leading to bacterial energy depletion [76]. Concurrently, phytol induces oxidative stress responses in *Pseudomonas aeruginosa* and directly disrupts bacterial DNA and protein synthesis, thereby blocking pathogen growth and replication at the molecular level [78].

Finally, as an antibacterial sensitizer, phytol demonstrates considerable potential for synergistic enhancement when combined with conventional antibiotics or other bioactive compounds (Figure 5D). Research indicates that phytol may enhance the bactericidal efficacy of ampicillin and ethidium bromide against *Staphylococcus aureus* by inhibiting bacterial efflux pump activity [79]. Furthermore, when co-administered with natural compounds such as β-sitosterol and stigmasterol, phytol exhibits a pronounced synergistic antibacterial effect against *Pseudomonas aeruginosa* [78]. Notably, semi-synthetic derivatives of phytol have been shown to reverse drug resistance in *Escherichia coli*, enhancing antibiotic efficacy by up to 16-fold, thereby offering a promising strategy to address clinical challenges associated with antimicrobial resistance [80].

### 4.4. Whitening Activity of Phytol

In addition to its notable antibacterial activity, phytol also demonstrates significant potential in skin cosmetology and therapeutics. Safety evaluations have confirmed that phytol is non-irritating to human skin in patch tests and exhibits no cytotoxicity in mouse melanoma cell models [10]. These findings provide a safety basis for its development as a depigmenting and whitening ingredient, as well as for the research and development of drugs targeting pigmentation-related skin disorders [10]. In regulating melanin biosynthesis, phytol exerts its hypopigmentary effects in cellular and enzymatic experimental models through a dual mechanism involving both modulation of cellular signaling pathways and direct inhibition of key biosynthetic enzymes [10,81].

Phytol effectively suppresses α-melanocyte-stimulating hormone (α-MSH)-induced melanogenesis in B16 melanoma cell models via activation of the ROS-ERK signaling cascade. Specifically, phytol moderately elevates intracellular ROS levels, which in turn induces phosphorylation of extracellular signal-regulated kinase (ERK). Activated ERK then promotes the ubiquitination and proteasomal degradation of microphthalmia-associated transcription factor (MITF). The subsequent downregulation of MITF leads to reduced expression of downstream melanogenic enzymes, including tyrosinase (TYR) and tyrosinase-related protein-1 (TRP-1), thereby inhibiting melanin synthesis at the transcriptional level [10].

In addition, phytol can directly inhibit tyrosinase activity in vitro [81]. Notably, its whitening efficacy can be further enhanced through structural modification. Previous studies have shown that esterified derivatives of phytol (e.g., phytol-4-methoxybenzoate) exhibit significantly stronger tyrosinase inhibitory activity than the parent compound [81]. This structure-activity relationship indicates that phytol is not only a potential whitening active molecule but also a crucial lead compound for developing novel, highly effective depigmenting agents.

### 4.5. Deep Repair: Anti-Aging Mechanisms and Maintenance of Skin Barrier Homeostasis

The anti-aging value of phytol lies not only in its direct matrix-protective effects but, more importantly, in its role as a mild “retinoid-like” precursor. By activating nuclear receptors through its metabolites, phytol achieves deep repair.

#### 4.5.1. Direct Anti-Aging Effects: Antioxidation, Matrix Protection, and Tissue Regeneration

Phytol exerts potential skin anti-aging effects in cellular models, molecular simulation studies and animal wound healing assays via multiple mechanisms. First, in terms of antioxidant defense, phytol exhibits superior free radical scavenging capacity compared to L-ascorbic acid. It effectively mitigates oxidative stress-induced damage to human keratinocytes (HaCaT cells), preserving cell membrane integrity, maintaining nuclear DNA stability, and safeguarding protein function. Consequently, it significantly suppresses cellular senescence phenotypes and delays skin aging [81,82]. Moreover, in UV-induced cellular photoaging models, both phytol and its structurally modified derivatives demonstrate notable protective effects, offering a novel strategy for developing high-efficiency, multi-target topical anti-aging formulations [63,83].

Second, in the context of extracellular matrix (ECM) protection, phytol maintains skin elasticity by targeting and regulating matrix metalloproteinases (MMPs). Molecular docking and molecular dynamics simulations confirm that phytol specifically binds to the catalytic domain of MMP-1, locking the enzyme’s active center through hydrogen bonding and hydrophobic interactions. This direct molecular inhibition effectively blocks MMP-1-mediated collagen degradation, fundamentally preventing the formation and deepening of skin wrinkles [84]. Finally, in tissue regeneration and repair, evidence from phytol-rich plant extracts suggests pro-regenerative potential. In an in-vitro fibroblast scratch-wound model, an ethanolic extract of *Anredera cordifolia*, which was reported to be predominantly composed of phytol in the specific extract batch used in that study, enhanced fibroblast migration and proliferation under both normal and hyperglycemic conditions, accompanied by elevated collagen production and up-regulated expression of ECM-related genes such as COL-1A and FGF-7 [85]. In summary, phytol achieves comprehensive intervention in skin aging through a tripartite mechanism: free radical scavenging, inhibition of matrix degradation, and promotion of tissue regeneration.

#### 4.5.2. Regulatory Mechanism of Phytanic Acid Metabolism

**Figure 6:**
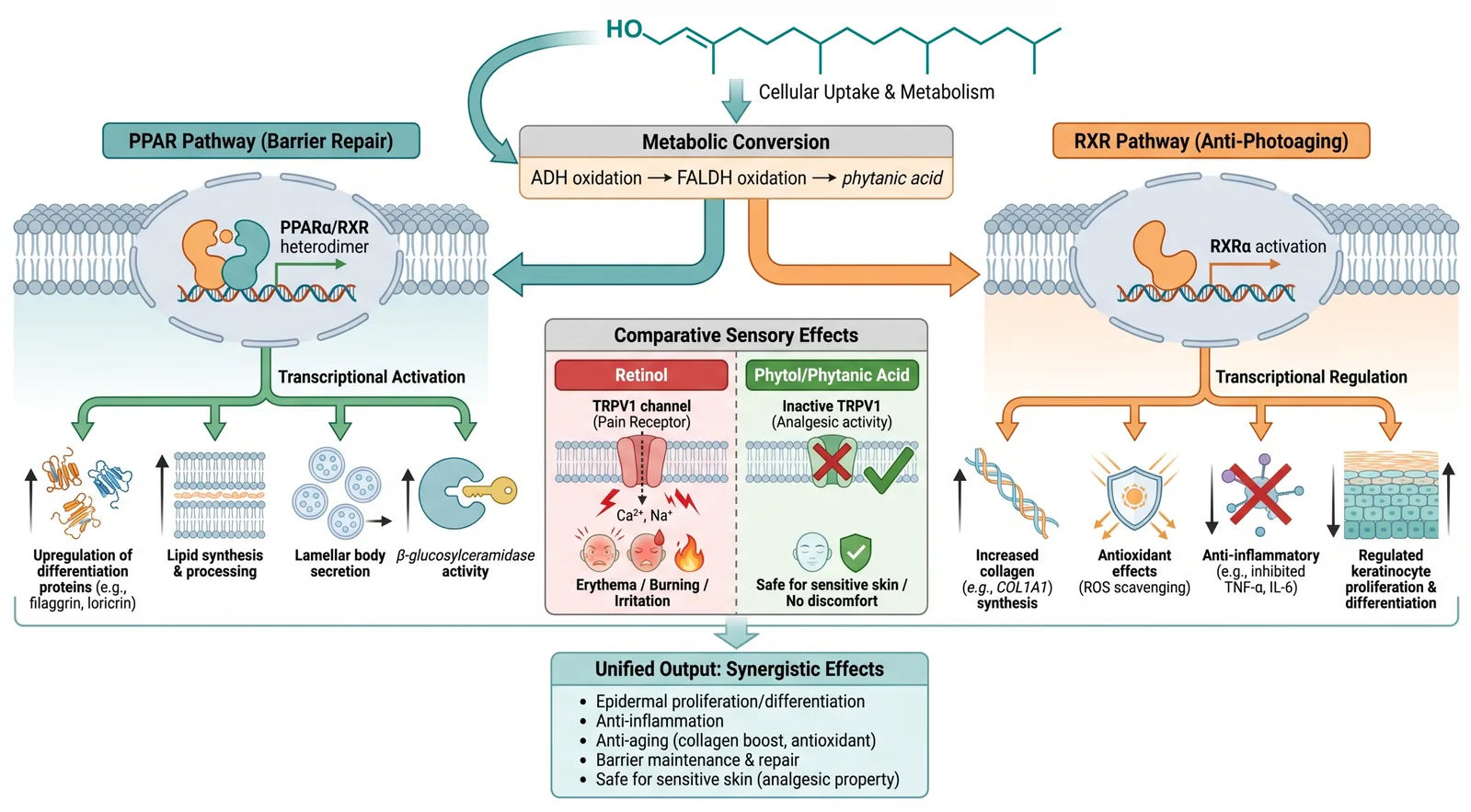
Mechanisms underlying the skin care effects of phytol. After cellular uptake, phytol is metabolized to phytanic acid via ADH/FALDH oxidation [44]. Phytanic acid activates the PPARα/RXR heterodimer to promote skin-barrier repair [87,88], up-regulate differentiation-related proteins, lipid metabolism and lamellar-body function [89]. It also activates RXRα to exert anti-photoaging effects [91,92], enhancing collagen production, antioxidant defense and anti-inflammatory responses, and controlling keratinocyte homeostasis. Unlike retinol which triggers TRPV1-mediated irritation [93], phytol/phytanic acid inactivates TRPV1 and shows analgesic and sensitive-skin-friendly properties [60]. Solid arrows represent activation/transcriptional up-regulation; crossed-block blunt arrows indicate channel or molecular inhibition. Colored boxes define distinct functional signaling modules. The central middle panel provides a direct comparative sensory-effect readout for retinol versus phytol/phytanic acid.

At the molecular level (Figure 6), it is generally hypothesized that phytol is first oxidized to phytenal by alcohol dehydrogenase (ADH) in the skin, and then further converted to phytanic acid via sequential enzymatic reactions including fatty aldehyde dehydrogenase (FALDH) catalysis [44,45]. Although ADH and FALDH family enzymes have been detected in human epithelial tissues including the epidermis, providing theoretical molecular prerequisites for this metabolic pathway within skin, direct experimental evidence regarding the metabolic conversion efficiency and intradermal metabolite accumulation of phytol in human skin remains insufficient, and relevant in-vivo validation is still required. Of note, once generated, phytanic acid has been experimentally demonstrated to act as a natural ligand that activates peroxisome proliferator-activated receptors (PPARs) and retinoid X receptors (RXRs) in a dose-dependent manner [86,87], thereby regulating critical skin physiological functions. Topical application of PPAR ligands promotes epidermal barrier repair, including upregulating the expression of differentiation-related proteins, enhancing barrier lipid synthesis, boosting lamellar body secretion, and increasing β-glucosylceramidase activity [88]. Notably, PPARα is predominantly expressed in the epidermis and is essential for maintaining barrier homeostasis by inducing keratinocyte differentiation, sustaining lipid production, and suppressing inflammation [89]. As a common dimerization partner of multiple nuclear receptors (including RARs and PPARs), RXRα activated by phytanic acid also plays a key role in skin homeostasis: its activation not only significantly increases collagen content and exerts antioxidant and anti-inflammatory effects but also regulates keratinocyte proliferation and differentiation following acute UV radiation [90,91].

#### 4.5.3. Safety Advantage: A Mild Functional Alternative to Retinoids

It should be clarified that there are essential differences in molecular targets between phytol and classic retinoids. Retinol and its derivatives mainly exert biological effects by binding to retinoic acid receptors (RARs) and forming RAR/RXR heterodimers, with strong regulatory effects on keratinocyte proliferation and collagen synthesis [92]. In contrast, phytanic acid, the metabolite of phytol, is known to act as a natural agonist of PPARs and RXRs [8,86]. Nevertheless, it remains hypothetical whether topically applied phytol can be sufficiently metabolized into phytanic acid within human skin to trigger these PPAR/RXR-mediated responses. If such metabolic conversion takes place, phytol may exert its effects predominantly via the PPAR/RXR signaling pathways [88,89]. Therefore, phytol may serve as a mild functional alternative to retinoids in skincare scenarios, rather than a complete retinoid agonist substitute. In terms of skin tolerance, phytol exhibits distinct safety advantages compared with retinol, the “gold standard” for clinical anti-aging. Retinol frequently causes adverse reactions such as erythema, burning, and stinging by activating the nociceptor TRPV1 [92,94,95]. In contrast, neither phytol nor its metabolites activate the TRPV1 channel; moreover, phytol itself possesses analgesic activity (Figure 6) [60], effectively bypassing neurogenic irritation. In summary, the putative phytol-phytanic acid system may represent one category of non-retinoid PPAR/RXR agonists [8], potentially delivering synergistic benefits including the promotion of epidermal proliferation and differentiation, anti-inflammation, anti-aging, and skin-barrier maintenance [9]. Accordingly, phytol could be considered a promising candidate for anti-aging applications targeting sensitive skin.

Compared with mature skincare active ingredients, phytol has a clear differentiated positioning. Compared with retinol, which has strong anti-aging efficacy but high skin irritation, phytol has lower irritation and better biocompatibility, but its single-component anti-aging intensity is weaker. Compared with vitamin E, which is a single antioxidant, phytol has a multi-dimensional effect covering anti-oxidation, anti-inflammation, barrier repair and whitening, with a wider range of applications. Compared with nicotinamide, which has high cost performance but certain intolerance risk, phytol has better tolerance and is more friendly to barrier-damaged skin. Compared with arbutin, which only targets whitening, phytol takes into account both soothing and repairing effects while inhibiting melanin, and is more suitable for sensitive skin with pigmentation problems.

Based on the reported properties of common skincare active ingredients, phytol has a clear differentiated positioning. Compared with retinol, which has strong anti-aging efficacy but high skin irritation, phytol has lower irritation and better biocompatibility, but its single-component anti-aging intensity is weaker. Unlike vitamin E, which is mainly used as an antioxidant, phytol exerts multi-dimensional effects covering anti-oxidation, anti-inflammation, barrier repair and whitening, with a wider range of applications. Relative to nicotinamide, which has high cost performance but certain intolerance risk, phytol has better tolerance and is more friendly to barrier-damaged skin. Compared with arbutin, which is mainly used for whitening, phytol takes into account both soothing and repairing effects while inhibiting melanin production, and is more suitable for sensitive skin with pigmentation problems.

### 4.6. Other Pharmacological Activities

Beyond its direct skin-related effects, phytol also exhibits systemic immunomodulatory and metabolic regulatory activities, primarily through the activation of PPAR and RXR nuclear receptors [93,96]. In terms of immunomodulation, phytol suppresses excessive inflammatory responses by inhibiting pro-inflammatory cytokine production and regulating macrophage polarization [97,98,99]. Regarding metabolic regulation, it improves lipid metabolism and insulin sensitivity via PPAR-mediated pathways, showing potential in ameliorating metabolic disorders such as dyslipidemia and insulin resistance [100,101,102]. Additionally, phytol has been reported to possess other bioactivities including neuroprotective and antitumor effects in various disease models [103,104]. These systemic effects, though not the focus of the present review, provide additional mechanistic support for the multi-target action of phytol in maintaining skin homeostasis, and may inspire the development of phytol-based skincare ingredients with both local and systemic benefits [105].

Collectively, numerous pre-clinical studies have described multiple biological properties of phytol, covering antioxidant, anti-inflammatory, antimicrobial, antimelanogenic and anti-ageing effects. These data are derived from heterogeneous experimental systems, including computational predictions, cell-based in vitro assays and diverse in vivo animal models, alongside indirect observations obtained from phytol-rich plant extracts.

Notably, there is a complete absence of published data using purified phytol in human-related studies. While multiple in vitro and animal studies have documented promising bioactivities of phytol, considerable translational gaps remain. Most observations are obtained under laboratory experimental conditions with dosing that may not reflect cosmetic-use concentrations; skin penetration, local skin safety, irritant potential, and real-world efficacy in human skin are still largely uncharacterized. In addition, in silico docking outputs represent computational predictions and require biological experimental validation. Some positive outcomes originate indirectly from complex plant extracts, where contributions from other co-existing constituents cannot be excluded. Therefore, promising pre-clinical findings cannot be directly extrapolated to practical cosmetic applications.

To clarify the current research deficits and guide further investigation, existing bottlenecks mainly lie in insufficient skin-related pharmacokinetic characterization, poorly defined safety and toxicity profiles, the lack of formulation-stability evaluation, and the absence of human-skin-relevant verification. Further targeted experiments focusing on skin-penetration assessment, dose optimization, safety validation, formulation-stability testing, and human skin-model verification are essential to advance the translational development of phytol for practical use.

To systematically compare the available evidence across different experimental platforms, Table 1 summarizes the main pharmacological activities and experimental evidence of phytol.

## 5. Limitations in Phytol Application

Despite its notable bioactivities, the broad application of phytol in cosmetics and pharmaceutical formulations is substantially limited by its intrinsic physicochemical properties. Specifically, the pronounced hydrophobicity of phytol results in poor aqueous dispersibility and a tendency to agglomerate in water-based systems, severely restricting uniform distribution and bioavailability in such formulations [11,12]. Moreover, its molecular structure is highly susceptible to light and heat, promoting oxidative degradation under common environmental conditions. This not only reduces the effective concentration of the active compound but may also adversely affect the sensory properties and safety profile of the final product [13]. These inherent limitations represent a critical bottleneck in the development of conventional phytol preparations, hindering precise delivery and controlled release, and thereby constraining its further development and use in high-efficacy skincare products and clinical therapeutics.

### 5.1. Formulation Strategies for Overcoming Phytol’s Application Limitations

To overcome the inherent physicochemical limitations of phytol, such as pronounced hydrophobicity and susceptibility to oxidative degradation, nano-drug delivery systems (NDDS) have been widely developed as a mainstream solution.

### 5.2. Permeation-Enhancing Properties and Formulation Basis of Phytol

Phytol, a terpenoid natural compound, functions as a natural penetration enhancer that effectively balances safety and efficacy. It demonstrates excellent transmembrane and transdermal permeation-promoting activity and is widely employed as a conventional chemical penetration enhancer (CPE) in cosmetic formulation design [106]. Its pronounced lipophilicity also ensures good compatibility with various oil-phase-based formulations, including creams, ointments, and nano-drug delivery systems, thereby providing a versatile basis for developing formulations tailored to different drug delivery scenarios. Recent studies have specifically designed composite ointment bases incorporating “phytol complexes” (concentrated phytol extracts), confirming the feasibility of phytol in topical drug delivery systems [107]. Furthermore, leveraging phytol’s ability to modulate lipid structures and enhance the absorption of active ingredients, researchers have developed a series of nano-drug carriers with phytol as a core functional component. Through computer-aided formulation optimization, phytol-based nanomicelles have been successfully prepared, significantly improving the cellular uptake efficiency of paclitaxel and purpurin-18 in cancer cells. This progress provides a promising technological pathway for the design of targeted antitumor delivery systems [108].

### 5.3. Strategies and Advances in Nano-Delivery Systems

To fully harness the biological activities of phytol, various nano-drug delivery systems have been developed and optimized, forming a synergistic and complementary framework.

Liposome-based systems utilize amphiphilic structure through coatings with zein or fucoidan, which not only significantly enhance the antioxidative stability of phytol but also improve its sustained release profile and anti-inflammatory delivery efficiency. Recent studies indicate that zein/fucoidan-coated phytol nanoliposomes maintain considerable DPPH radical-scavenging activity during sustained in vitro release, with encapsulation substantially improving both antioxidative stability and prolonged activity of phytol [109]. Phytol’s intrinsic anti-inflammatory activity is notably amplified when delivered via liposomal systems [110]. In vivo studies further confirm that liposomal delivery enhances phytol’s efficacy in carrageenan-induced paw edema models [111].

Nanoemulsions, as a thermodynamically stable system, significantly enhance the solubility and dispersibility of phytol. When delivered via soybean oil-based nanoemulsion carriers, phytol exhibited stronger inhibitory activity against *Leishmania amazonensis* promastigotes. The 24-h IC_50_ value of free phytol was 480.5 μg/mL, whereas the phytol-loaded nanoemulsion achieved a lower IC_50_ of 289.7 μg/mL, demonstrating that nanoformulation markedly improved antiprotozoal potency while maintaining lower cytotoxicity, providing a stable platform for topical and transdermal applications [112]. Furthermore, alginate hydrogel bead-encapsulated phytol nanoemulsions provide effective protection and therapeutic efficacy against alcohol-induced gastric ulcers by modulating the nitric oxide (NO) synthase pathway, suppressing inflammatory responses, and upregulating the expression of transforming growth factor-β1 (TGF-β1) [113]. Notably, this case illustrates the stabilizing and anti-inflammatory merits afforded by nanoencapsulation of phytol. In addition, nanoemulsion formulations substantially enhance the antioxidant capacity of phytol, achieving significantly higher scavenging rates against DPPH•, ABTS•+, •OH, and NO• radicals compared to free phytol [57].

Poly (lactic-co-glycolic acid) (PLGA) nanoparticles leverage good biodegradability and controlled-release properties for targeted delivery of phytol, demonstrating notable efficacy in alleviating cognitive dysfunction associated with neurodegenerative disorders. Phytol-loaded PLGA nanoparticles effectively ameliorate scopolamine-induced cognitive impairment in Wistar rats by attenuating cholinesterase activity, oxidative stress, and apoptotic pathways [114]. They also counteract amyloid beta-induced neurotoxicity in cellular and transgenic *Caenorhabditis elegans* models through the regulation of Alzheimer’s disease-related gene expression and inhibition of neuronal apoptosis [115]. These findings underscore the controlled-release and antioxidant properties of phytol-loaded nanocarriers.

Solid lipid nanoparticles (SLNs), which encapsulate phytol within solid lipid matrices, effectively overcome its poor aqueous solubility in antifungal applications. These systems significantly enhance antifungal efficacy and promote prolonged skin retention. Phytol-loaded SLNs have been demonstrated to substantially lower the minimum inhibitory concentration (MIC) of phytol against 15 strains of Candida species [116]. Meanwhile, nanoemulsified phytol readily penetrates microbial biofilms, improving its bioavailability [117]. In transdermal systems, rationally designed phytol-loaded SLNs exhibit particle size and stability suitable for skin permeation, reducing safety risks, enhancing stratum corneum penetration, and enabling sustained drug retention and controlled release, thereby improving transdermal bioavailability [118].

These nanocarrier systems possess distinct strengths and drawbacks, and may potentially complement each other for various scenarios, forming a multi-tiered delivery platform for phytol-related applications [119]. In summary, nanocarrier technology not only overcomes the skin barrier via the “small-size effect” but also fundamentally resolves phytol’s physicochemical limitations—hydrophobicity and oxidative instability—offering a robust theoretical and technical foundation for its advanced use in high-efficacy skincare products and clinical therapeutics. Building on these established nano-delivery strategies, phytol has gradually transitioned from laboratory research to industrial translation, and has been incorporated into a growing number of commercial skincare products worldwide.

To systematically review the research progress of phytol-based nanocarriers, Table 2 summarizes the relevant literature on phytol-based nanocarriers. The table covers multiple carrier types, including liposomes, nanoemulsions, PLGA nanoparticles, and solid lipid nanoparticles (SLNs), and presents a comparative analysis of key indicators such as particle size, polydispersity index (PDI), zeta potential, encapsulation efficiency (EE, %) and release duration, highlighting their enhanced stability, bioavailability, and functional advantages over free phytol.

## 6. Market Applications of Phytol

Relying on its mild, multi-effect characteristics and differentiated positioning as a gentle alternative to retinoids, phytol has been gradually applied in mid-to-high-end skincare products focusing on sensitive skin repair and mild anti-aging. Leveraging its notable antioxidant, anti-aging, anti-inflammatory, and transdermal penetration-enhancing properties, phytol has demonstrated significant potential in skincare applications [7]. Importantly, it offers considerable advantages over conventional retinol in terms of safety and formulation compatibility [94]. According to the EU CosIng cosmetic-ingredient database maintained by the European Commission, phytol is catalogued as an active cosmetic ingredient with skin-conditioning-emollient function [121]. As a promising functional cosmetic ingredient, phytol can be incorporated into composite anti-aging systems together with retinoid-related substances. Such combined systems may retain anti-aging potency while alleviating retinol-triggered cutaneous irritation, which provides a potential formulation strategy for anti-aging skincare tailored to sensitive skin types.

## 7. Conclusions and Prospects

As a natural terpenoid compound, phytol possesses excellent biological activities including antibacterial, antioxidant, anti-inflammatory, and anti-aging properties, showing great potential in skincare development. Current studies on phytol are still dominated by fundamental in vitro and animal explorations, and phytol is more commonly adopted as a simple cosmetic additive instead of a well-validated functional skincare ingredient, lacking a complete and feasible translational pathway from laboratory research to standardized industrial skincare products.

Notably, most reported pharmacological and mechanistic evidence is derived from non-skin-specific models, including neuronal, skeletal, gastrointestinal and systemic disease systems, which only reflects the general pharmacological potential of phytol rather than confirming direct topical skincare efficacy. In addition, most existing studies focus on improved solubility, antioxidant capacity, sustained release and antibacterial performance, while direct validations based on Franz diffusion cells, ex vivo skin penetration models and intradermal drug retention quantification that reflect real skin bioavailability remain scarce. Consistently, nearly all skincare-related bioactivity assessments are limited to preclinical cell and rodent experiments, with a complete absence of large-sample human clinical data, making the actual in-vivo efficacy and long-term skin safety of topical phytol formulations insufficiently verified.

Although phytol is considered a mild retinoid-like candidate with lower acute irritation compared with conventional retinoids, supporting its potential suitability for sensitive-skin applications, current safety evaluations remain incomplete. Available evidence lacks systematic assessment of repeated-use irritation, skin sensitization, phototoxicity, photosensitization, oxidative metabolite risks, concentration-dependent cytotoxicity, and long-term skin barrier homeostasis effects. Clear safe concentration ranges without adverse cutaneous reactions have not yet been fully established.

Furthermore, the practical translation of phytol is severely restricted by its poor aqueous solubility, insufficient photothermal stability, and unclear local metabolic fate in human skin. Current nanocarrier encapsulation strategies mainly improve its physicochemical properties, while skin-targeted delivery optimization, skin-specific pharmacokinetic characteristics, in-situ metabolic regulation and dose-effect relationships remain poorly clarified. Importantly, the hypothetical metabolic conversion of phytol to phytanic acid within human skin and its direct contribution to skincare efficacy still lack definitive in-vivo validation, representing a core mechanistic gap.

To address the above translational and mechanistic limitations, future research should focus on concrete and prioritized directions rather than general explorations. These include the establishment of standardized phytol formulation systems, systematic skin-related pharmacokinetic and bioavailability evaluation via ex vivo skin penetration models, targeted validation of predicted PPAR/RXR regulatory mechanisms, comprehensive concentration-gradient safety and toxicity assessment, optimization of cosmetic-compatible dosage ranges, scaled-up manufacturing verification, and prerequisite human skin model and volunteer tests to support subsequent clinical trials. Moreover, innovative delivery strategies utilizing phytol itself as a penetration-enhancing carrier backbone deserve further in-depth exploration, which may provide new insights for the development of high-efficiency phytol-based multi-functional skincare systems.

## Figures and Tables

**Table 1:** Summary of main pharmacological activities and experimental evidence of phytol.

Main Pharmacological Activities	Research Type	Key Findings
Antioxidant	In vitro [55,56,57,58,59,65]	Phytol directly scavenges DPPH•, •OH, and NO•, enhances SOD/CAT activities, and elevates GSH content, thereby attenuating intracellular ROS accumulation. (Direct) Phytol exerts antioxidative cytoprotection via Nrf2/HO-1-dependent responses in cell-based models; it inhibits lipid peroxidation in TBARS assays. (Direct) Phytol was identified by GC-MS as a constituent of *Betula pendula* leaf extract; the extract exhibits antioxidant activity in DPPH and FRAP assays. (Indirect)
	In vivo [55,57]	In Swiss mice, intraperitoneal phytol (25–75 mg/kg) dose-dependently reduced hippocampal lipid peroxidation and nitrite levels while increasing SOD, CAT, and GSH activities. (Direct) In *S. cerevisiae* oxidative stress models, phytol and its nanoemulsion protected yeast cells against H_2_O_2_-induced damage. (Direct)
Antinociceptive and Antipruritic	In vitro [69]	Phytol, identified as a principal component of the bioactive extract fraction, contributes to LOX-inhibitory and anti-inflammatory effects in RAW264.7 cells, thereby providing indirect evidence for its antinociceptive and antipruritic properties. (Indirect)
	In vivo [56,60,61,69]	Phytol exhibits antinociceptive activity validated in multiple models (writhing/formalin/hot plate) without motor impairment. (Direct) Attenuation of CFA-induced hyperalgesia by phytol occurs via the p38MAPK/NF-κB pathway, alongside inhibition of histamine- and compound 48/80-triggered scratching. (Direct) Phytol is a major constituent of the active extract fraction; the extract gel promotes wound healing in a mouse excisional wound model. (Indirect)
Anti-inflammatory	In silico [64]	Molecular docking suggests phytol targets key anti-inflammatory enzymes such as COX-2, with the strongest binding affinity for this isoform. (Direct)
	In vitro [60,62,64,70]	Phytol promotes macrophage M2 polarization and reduces proinflammatory cytokines in primary human immune cells. (Direct) Dose-dependent inhibition of protein denaturation is observed for phytol in in vitro assays. (Direct) Phytol-containing essential oil reduces IL-1β/IL-6/TNF-α/PGE_2_/NO and inhibits iNOS/COX-2 and the MAPK/NF-κB pathway in RAW264.7 cells. (Indirect)
	In vivo [62,64,71]	Paw edema in multiple models (carrageenan/formalin, etc.) is inhibited by phytol, which attenuates CFA-induced arthritis via the p38MAPK/NF-κB pathway. (Direct) Neutrophil migration is suppressed by phytol, which shows synergistic effects with NSAIDs. (Direct) Phytol-containing essential oil applied topically attenuates xylene-induced ear swelling in mice and decreases proinflammatory cytokines in tissues and serum. (Indirect)
Antimicrobial	In silico [75,78,79]	Molecular docking shows strong binding of phytol to KPC-2/OXA-48 carbapenemases; hydrogen bonding and efflux pump interactions support resistance reversal and antibiotic potentiation. (Direct) Phytol is a major constituent of the antibacterial active fraction of *Pulicaria crispa*; the fraction was docked against PBP2A/DNA gyrase B but phytol was not validated in isolation. (Indirect)
	In vitro [72,73,74,75,76,77,78,79]	Phytol exerts direct antibacterial activity against *E. coli*, *C. albicans*, *A. niger*, and carbapenem-resistant *K. pneumoniae*, with weak activity against *S. aureus*. (Direct) Phytol achieves bactericidal effects via membrane disruption and ROS induction, and enhances the efficacy of norfloxacin/gentamicin against drug-resistant bacteria. (Direct) Phytol-containing extracts/essential oils show antibacterial activity against diverse bacteria, fungi, and drug-resistant strains such as MRSA; mechanisms include membrane disruption, respiratory enzyme inhibition, and downregulation of PBP2A/DNA gyrase B. (Indirect)
	In vivo [72]	Topical cream of phytol-containing *Arthrospira platensis* extract effectively treats cutaneous *C. albicans* infection in mice. (Indirect)
Whitening Activity	In silico [81]	Molecular docking shows phytol derivatives bind tyrosinase more strongly than phytol; structural modifications enhance active-site interactions. (Direct)
	In vitro [10]	Phytol dose-dependently inhibits α-MSH-induced melanogenesis, outperforming arbutin, kojic acid, and resveratrol without cytotoxicity. (Direct) Phytol promotes MITF degradation via the ROS-ERK pathway, thereby downregulating tyrosinase and TRP1. (Direct)
Anti-Aging Maintenance of Skin Barrier Homeostasis	In silico [84]	Molecular docking shows phytol binds MMP-1 via a hydrogen bond with His132, suggesting anti-wrinkling potential. (Direct)
	In vitro [81,82]	Phytol dose-dependently inhibits H_2_O_2_-induced senescence in HaCaT keratinocytes with no cytotoxicity; it activates Nrf2/HO-1, reduces inflammatory factors, and inhibits apoptosis. (Direct) Inhibition of ECM-degrading enzymes including elastase, collagenase and hyaluronidase points to anti-wrinkling potential upon phytol treatment. (Direct)

**Table 2:** Physicochemical profiles and advantages of phytol-based nanocarriers.

Nanocarrier Type	Characterization of Nanoparticle	Key Advantages over Free Phytol
Liposomes [109,111]	Size: 142.14 ± 8.66 nm PDI: 0.24 ± 0.01 Zeta: −74.93 ± 1.10 mV EE: 76.19 ± 0.03% Release Duration: 6 h (120 min SGF + 240 min SIF) Experimental Model: 1. In vitro simulated GI digestion (SGF/SIF) 2. TBHP-induced oxidative stress in HepG2 cells	Prevent premature gastric release of phytol Higher antioxidant activity Better protection against TBHP-induced HepG2 oxidative damage Reduced cytotoxicity at equal phytol dosage Improved physicochemical stability
	Size: 263.1 ± 4.5 nm PDI: N/A Zeta: N/A EE: 88.15 ± 2.67% Release Duration: N/A Experimental Model: 1. carrageenan-induced paw edema in rats 2. AML and leukemia cell lines	Stronger anti-inflammatory effect against carrageenan-induced paw edema Higher anticancer potency at lower concentrations for specific leukemia cell lines Improve poor aqueous-solubility of phytol
Nanoemulsions [112,113,117]	Size: ~210 nm PDI: <0.2 Zeta: ~−20 mV EE: N/A Release Duration: N/A Experimental Model: 1. 3T3 fibroblast-like cells 2. *Leishmania amazonensis*	Higher antileishmanial potency with lower IC_50_ value Reduced cytotoxicity towards mammalian 3T3 fibroblasts Improved colloidal stability and lipophilic phytol dispersibility
	Size: 71.69 ± 4.68 nm PDI: 0.15 ± 0.040 Zeta: −15.27 ± 0.61 mV EE: 88.67 ± 0.85% Release Duration: pH 6.8 (SIF): ~50% released at 8 h, equilibrium ~87% at 12 h pH 1.2 (SGF): ~20% released by end of experiment Experimental Model: 1. In vitro: USP Type II apparatus, SGF (pH 1.2)/SIF (pH 6.8), 37 °C 2. In vivo: Male Wistar rats, ethanol-induced acute gastric ulcer	Enhanced anti-ulcer activity against ethanol-induced gastric injury Improved penetration into multi-layers of gastric mucosa PH-dependent sustained release, reduced premature gastric release Improved aqueous solubility and formulation stability
	Size: 245.50 ± 25.02 nm PDI: 0.384 Zeta: −77.8 ± 2.83 mV EE: N/A Release Duration: N/A Experimental Model: *Artemia salina*; *Allium cepa*	Enhanced cellular uptake and bioavailability Stronger cytotoxic and genotoxic effects at high concentrations Similar adaptive DNA-damage-preventing capacity at low concentrations Improved aqueous solubility of lipophilic phytol
PLGA Nanoparticles [114,115,120]	Size: 177.4 ± 5.9 nm PDI: 0.2 ± 0.06 Zeta: −32.8 ± 2.2 mV EE: 92.14 ± 0.29% Release Duration: Biphasic: ~22% burst release within 6 h, followed by sustained release reaching ~98% cumulative release at 120 h Experimental Model: 1. Neuro-2a cells 2. Wistar rats (scopolamine-induced amnesia)	Enhanced blood-brain-barrier penetration and brain biodistribution Sustained biphasic drug release profile Superior amelioration of scopolamine-induced cognitive impairment Stronger inhibition of Aβ aggregation, cholinesterase and oxidative stress Improved aqueous solubility of lipophilic phytol
	Size: 189.3 ± 5 nm PDI: 0.224 Zeta: 0.1 mV EE: 8.43 ± 0.01% Release Duration: 120 h; sustained release with cumulative release up to 98.4%; biphasic pattern (initial burst followed by sustained release) Experimental Model: 1. A549 human lung adenocarcinoma cells 2. L-132 normal human lung epithelial cells 3. Zebrafish (*Danio rerio*) embryos	Improved aqueous solubility of lipophilic phytol Sustained biphasic in-vitro drug release Enhanced anti-proliferative and pro-apoptotic effect against A549 cells Higher selectivity towards cancer cells, reduced toxicity to normal L-132 cells Good biosafety in zebrafish-embryo toxicity assay
Solid Lipid Nanoparticles (SLN) [116,118]	Size: 302.2 ± 10.0 nm PDI: 0.12 ± 0.03 Zeta: −16.7 ± 0.5 mV EE: 68.74% Release Duration: N/A Experimental Model: 1. HEK-293 cells 2. 15 *Candida* spp. Strains	Improved aqueous dispersibility of lipophilic phytol Greatly enhanced anticandidal activity with much lower MIC values Promoted penetration across fungal cell wall and membrane Good colloidal stability during long-term storage Higher cytotoxicity to mammalian cells at elevated concentrations
	Size: 292.29 ± 6.37 nm PDI: 0.16 ± 0.03 Zeta: −25.4 ± 1.01 mV EE: 89.20% Release Duration: ~600 min (10 h); sustained release with ~47% phytol released Experimental Model: Vero cells	Improved aqueous dispersibility of lipophilic phytol Achieved sustained-slow in-vitro drug release Enhanced thermal stability of encapsulated phytol Good colloidal stability during long-term storage Comparable cytotoxicity against Vero cells

## Data Availability

No new data were created or analyzed in this study. Data sharing is not applicable to this article.
